# Acute Spinal Cord Injury: Correlations and Causal Relations Between Intraspinal Pressure, Spinal Cord Perfusion Pressure, Lactate-to-Pyruvate Ratio, and Limb Power

**DOI:** 10.1007/s12028-020-00988-2

**Published:** 2020-05-20

**Authors:** Florence R. A. Hogg, Siobhan Kearney, Argyro Zoumprouli, Marios C. Papadopoulos, Samira Saadoun

**Affiliations:** 1 Academic Neurosurgery Unit, St. George’s, University of London, London, UK; 2 Neuroanaesthesia/Neuro Intensive Care, St. George’s Hospital, London, UK

**Keywords:** Blood pressure, Intraspinal pressure, LPR, Management, Microdialysis, Monitoring, Spinal cord injury

## Abstract

**Background/Objective:**

We have recently developed monitoring from the injury site in patients with acute, severe traumatic spinal cord injuries to facilitate their management in the intensive care unit. This is analogous to monitoring from the brain in patients with traumatic brain injuries. This study aims to determine whether, after traumatic spinal cord injury, fluctuations in the monitored physiological, and metabolic parameters at the injury site are causally linked to changes in limb power.

**Methods:**

This is an observational study of a cohort of adult patients with motor-incomplete spinal cord injuries, i.e., grade C American spinal injuries association Impairment Scale. A pressure probe and a microdialysis catheter were placed intradurally at the injury site. For up to a week after surgery, we monitored limb power, intraspinal pressure, spinal cord perfusion pressure, and tissue lactate-to-pyruvate ratio. We established correlations between these variables and performed Granger causality analysis.

**Results:**

Nineteen patients, aged 22–70 years, were recruited. Motor score versus intraspinal pressure had exponential decay relation (intraspinal pressure rise to 20 mmHg was associated with drop of 11 motor points, but little drop in motor points as intraspinal pressure rose further, *R*^2^ = 0.98). Motor score versus spinal cord perfusion pressure (up to 110 mmHg) had linear relation (1.4 motor point rise/10 mmHg rise in spinal cord perfusion pressure, *R*^2^ = 0.96). Motor score versus lactate-to-pyruvate ratio (greater than 20) also had linear relation (0.8 motor score drop/10-point rise in lactate-to-pyruvate ratio, *R*^2^ = 0.92). Increased intraspinal pressure Granger-caused increase in lactate-to-pyruvate ratio, decrease in spinal cord perfusion, and decrease in motor score. Increased spinal cord perfusion Granger-caused decrease in lactate-to-pyruvate ratio and increase in motor score. Increased lactate-to-pyruvate ratio Granger-caused increase in intraspinal pressure, decrease in spinal cord perfusion, and decrease in motor score. Causality analysis also revealed multiple vicious cycles that amplify insults to the cord thus exacerbating cord damage.

**Conclusion:**

Monitoring intraspinal pressure, spinal cord perfusion pressure, lactate-to-pyruvate ratio, and intervening to normalize these parameters are likely to improve limb power.

**Electronic supplementary material:**

The online version of this article (10.1007/s12028-020-00988-2) contains supplementary material, which is available to authorized users.

## Introduction

Traumatic spinal cord injury (TSCI) affects about 180,000 people globally each year [1]. Prognosis is generally poor. For example, more than a third of patients have paraplegia or quadriplegia, fewer than 1% are discharged neurologically normal and life expectancy is below national averages [2]. No treatment for acute TSCI has been proven to improve outcome: the effects of early surgery [3–7], intravenous methylprednisolone [8–12] or maintaining mean arterial pressure (MAP) 85–90 mmHg for a week after TSCI [13–16] remain controversial [17].

To improve the management of patients with TSCI in the intensive care unit (ICU), multi-modality monitoring from the injury site has been suggested [18]. Intraspinal pressure (ISP) and spinal cord perfusion pressure (SCPP) [19–21] are monitored with a pressure probe, as well as injury site metabolism with a microdialysis (MD) catheter [22, 23]. These techniques are safe and analogous to multi-modality monitoring for brain injury [24]. ISP and SCPP correlate with injury site metabolism [25], neurological status [26], and long-term neurological outcome [20], and patients with neurologically complete TSCIs have more deranged cord metabolism than those with neurologically incomplete injuries [22]. These relations between ISP, SCPP, and injury site metabolism versus neurological outcome are associations that do not imply causation.

The objective of our study is to address the question whether changes in ISP, SCPP, and injury site metabolism *cause* changes in limb power. This is paramount because it implies that interventions to normalize ISP, SCPP, and injury site metabolism would improve limb power. To determine causation, we focused on grade C (American Spinal Injury Association Impairment Scale, AIS) injuries because they have some intact limb power below the injury. Limb motor score, ISP, SCPP, and injury site lactate-to-pyruvate ratio (LPR) were monitored for up to a week after surgery. We used these time series to investigate the hypothesis that fluctuations in motor score are caused by fluctuations in the physiology and metabolism at the injury site. We employed the concept of causality proposed by Clive Granger [27] and recently reviewed [28], i.e., a variable, such as SCPP, which evolves in time, Granger-causes another time-evolving variable, such as motor score, if predictions of motor score based on its own past and on the past of SCPP are better than predictions of motor score based solely on its own past.

## Methods

### Institutional Research Board Approvals

Injured Spinal Cord Pressure Evaluation (ISCoPE) is a clinical study at St. George’s Hospital in London, U.K., registered at www.clinical trials.gov as NCT02721615. Approvals for the ISCoPE study including the consent form and patient information sheet were obtained by the St. Georges Joint Research Office and the U.K. National Research Ethic Service—Camberwell St Giles Committee (No 10/H0807/23). The study has been performed in accordance with the ethical standards as laid down in the 1964 Declaration of Helsinki and its later amendments. Informed consent was obtained from all individual participants included in the study.

### Study Design

We investigated the correlations and causal relations between ISP, SCPP, tissue LPR, glutamate, and standardized motor score in AIS C TSCI patients.

### Inclusion/Exclusion Criteria

We selected all AIS grade C TSCI patients who were enrolled into ISCoPE for the period October 2011–January 2020. Inclusion criteria for ISCoPE are: severe TSCI grades A–C, age 18–70 years and surgery performed within 72 h of injury. Exclusion criteria are: major co-morbidity, inability to obtain consent, and penetrating TSCI. The ISCoPE study initially aimed to develop ISP monitoring from the injury site. In 2014, we also started monitoring MD. The current study includes AIS grade C patients who only had ISP monitoring (recruited 2011–4) as well as those who also had MD monitoring (recruited 2014–2020).

### Clinical Examination and Imaging

All patients were admitted to the neurosurgical unit at St. George’s Hospital and underwent International Standards for Neurological Classification of Spinal Cord Injury assessments by a trained neurosurgical resident, which was repeated at discharge and in follow-up clinic. Postoperatively, all patients were admitted to the Neuro-ICU. As standard clinical care in our unit, all TSCI patients undergo regular motor limb assessments by nurses trained in Medical Research Council grading of limb power. These assessments were performed with the patient off sedation or during a sedation hold and recorded in spinal assessment charts. Patients had CT and MRI of the spine before surgery and within four weeks of surgery.

### Probe Insertion

**Fig. 1:**
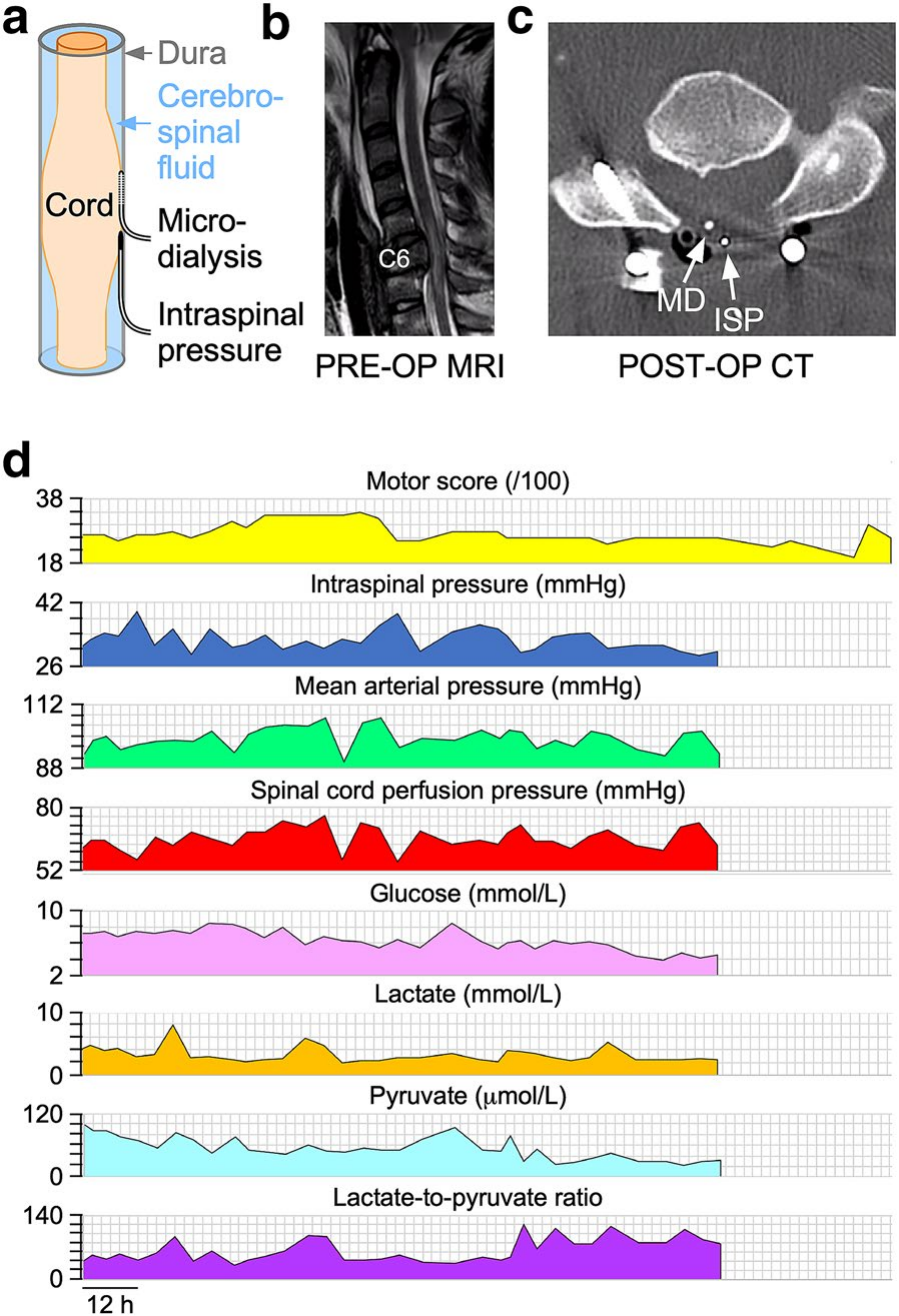
Monitoring setup. **a** Intraspinal pressure probe and microdialysis catheter inserted intradurally to monitor from injured cord. **b** Preoperative sagittal T2 MRI of patient no. 52 with spinal cord injury at C6. **c**. Postoperative axial CT with intraspinal pressure probe and microdialysis catheter in situ. **d** Multi-modality monitoring of motor score (yellow), intraspinal pressure (blue), mean arterial pressure (green), spinal cord perfusion pressure (red) as well as tissue glucose (pink), lactate (orange), pyruvate (cyan), and lactate-to-pyruvate ratio (purple) (Color figure online)

Surgical decompression and spinal instrumentation were performed by a neurosurgeon based on patient requirements and surgeon preference. Surgical decompression was laminectomy with or without corpectomy with spinal instrumentation based on surgeon preference. Posterior fixation was with lateral mass screws for the cervical spine and pedicle screws for the thoracic spine. Anterior cervical fixation was with vertebral body plate and screws. During the posterior approach, a pressure probe (Codman Microsensor Transducer®, Depuy Synthes, Leeds, UK) and a MD catheter (CMA61: CMA microdialysis AB, Solna, Sweden) were placed intradurally on the surface of the injured cord at the site of maximal cord swelling based on the preoperative MRI (Fig. 1). The dural opening was sutured and supplemented with fibrin glue (Tisseel®, Baxter, UK). Postoperative CT with the probes in situ confirmed positioning.

### Intraspinal Pressure and Blood Pressure Monitoring

The pressure probe was connected to a Codman ICP box linked via a ML221 amplifier to a PowerLab running LabChart v.8 (AD Instruments, Oxford, UK). Blood pressure was recorded from a radial artery catheter connected to the Philips Intellivue MX800 bedside monitoring system (Philips, Guildford, UK), in turn connected to the PowerLab system. ISP and blood pressure signals were sampled at 1 kHz, and patients were monitored for up to a week. Data were analyzed using Labchart version 8 (AD Instruments, Oxford, UK) and ICM+ (www.neurosurg.cam.ac.uk/icmplus). We computed SCPP as MAP–ISP. ISP is different from intrathecal pressure measured above or below the injury because the swollen, injured cord is compressed against the dura thus compartmentalizing the intrathecal space as illustrated in Fig. 1a and described in earlier publications [29–31].

### Microdialysis Setup and Analysis

MD was started postoperatively in the Neuro-ICU as described [22, 23, 25]. Central nervous system fluid (CMA microdialysis AB) was perfused at 0.3 μL/min using the CMA106 pump (CMA microdialysis AB). MD vials were changed hourly and analyzed using ISCUS Flex (CMA microdialysis AB) for glucose, lactate, and pyruvate. The LPR was calculated. The first two samples from each patient were discarded to allow priming of the MD catheter and stabilization of the metabolite concentrations. 100-fold changes in metabolite concentration, compared with the preceding hour, were excluded from analysis. Metabolite levels were compared to corresponding hourly averages of ISP, MAP, and SCPP. Our MD method measures spinal cord surface metabolism at the injury site, which correlates with intraparenchymal injury site metabolism, but is different from metabolites measured from lumbar cerebrospinal fluid [22, 23, 32].

### Standardization of Limb Motor Score

The motor scores in the spinal assessment charts were standardized by subtracting the minimum postoperative motor score for each patient from all motor scores for that patient. Standardized motor scores were compared to ISP, MAP, SCPP, and MD values averaged over the hour corresponding to the neurological assessment.

### Summary of Variables/Outcomes

We obtained matched values of ISP, SCPP, tissue glucose, LPR, and standardized motor score. To avoid bias, data were analyzed blindly, i.e., each variable/outcome was obtained and listed for analysis without knowledge of the other associated variables.

### Statistical Analysis

Motor score (*y*) versus ISP (*x*) was fitted with an exponential decay curve $y=A+B{e}^{-Cx}$, motor score versus SCPP and versus LPR were fitted with linear equations, whereas motor score (*y*) versus MAP (*x*) was fitted with a bounded exponential curve $y=A+B(1-{e}^{-Cx})$ using the online free curve fitting service (https://mycurvefit.com/). Plots are mean ± standard error. For Granger causality analysis of the standardized motor score, ISP, SCPP, and LPR time series, we used the Granger function in the Cloud Causal Analytics Toolkit (https://cox-associates.com/) that employs vector autoregression models to test for causal relations. For details, see Supplement.

## Results

### Participants

There were 19 patients, with average age 47 years (range 19–70), of which 14 (74%) were male and 5 (26%) female. Fourteen (74%) had cervical and 5 (26%) thoracolumbar injuries. Of the 19 patients, 12 (63%) had posterior surgical approach plus laminectomy, 5 had anterior plus posterior approach and laminectomy (26%) and 2 (11%) had posterior approach without laminectomy. Mean time from injury to surgery was 38 h. All 19 patients had ISP monitoring and 13 (68%) also had MD monitoring. The mean duration of ISP monitoring was 5.1 days (range 1.8–6.8) and of MD monitoring 3.7 days (range 0–6.8). The mean number of motor exams in the monitored period was 19 (range 5–37). Table 1 has details.

**Table 1:** Demographic details of the AIS grade C patients

Pt. No	Study no	Age (y)	Sex	Level	Surgical approach	Lami	Injury to surgery (h)	ISP monitoring (h)	MD monitoring (h)	No. of motor exams
1	8	64	F	C5	Post	No	47	94	0	6
2	9	49	F	C5	Post	Yes	16	43	0	6
3	10	54	M	T12	Post	Yes	48	117	0	13
4	13	65	M	C4	Post + Ant	Yes	11	68	0	10
5	20	59	M	C6	Post	Yes	32	71	0	14
6	40	70	F	C4	Post	Yes	20	156	156	23
7	44	26	M	T12	Post	Yes	46	160	35	23
8	45	37	M	C4	Post + Ant	Yes	29	136	138	25
9	50	63	M	C7	Post	Yes	47	105	0	12
10	52	19	M	C6	Post	Yes	41	145	145	28
11	54	63	M	C5	Post	Yes	58	138	137	25
12	61	56	F	L1	Post	Yes	31	114	161	27
13	66	67	M	C4	Post	No	38	160	157	25
14	67	32	M	C4	Post + Ant	Yes	22	159	163	35
15	68	37	F	L3	Post	Yes	23	139	125	22
16	70	35	M	C4	Post + Ant	Yes	39	112	109	5
17	71	27	M	L1	Post	Yes	41	144	145	37
18	81	54	M	C4	Post	Yes	69	161	150	25
19	84	22	M	C6	Post + Ant	Yes	70	84	82	5

*Ant* anterior, *C* cervical, *F* female, *h* hours, *Lami* laminectomy, *L* lumbar, *M* male, *No*. number, *Post* posterior, *Pt.* patient, *T* thoracic, *y* years

### Complications

Two patients (11%) had cerebrospinal fluid leak from around the probe skin exit site which stopped with additional sutures, and 4 patients (21%) had asymptomatic pseudomeningocele on the postoperative MRI. Four patients (21%) had chest sepsis, and 1 (5.3%) patient developed Korsakoff psychosis from alcohol withdrawal.

### Motor Score Strongly Correlates with Injury Site Physiology

**Fig. 2:**
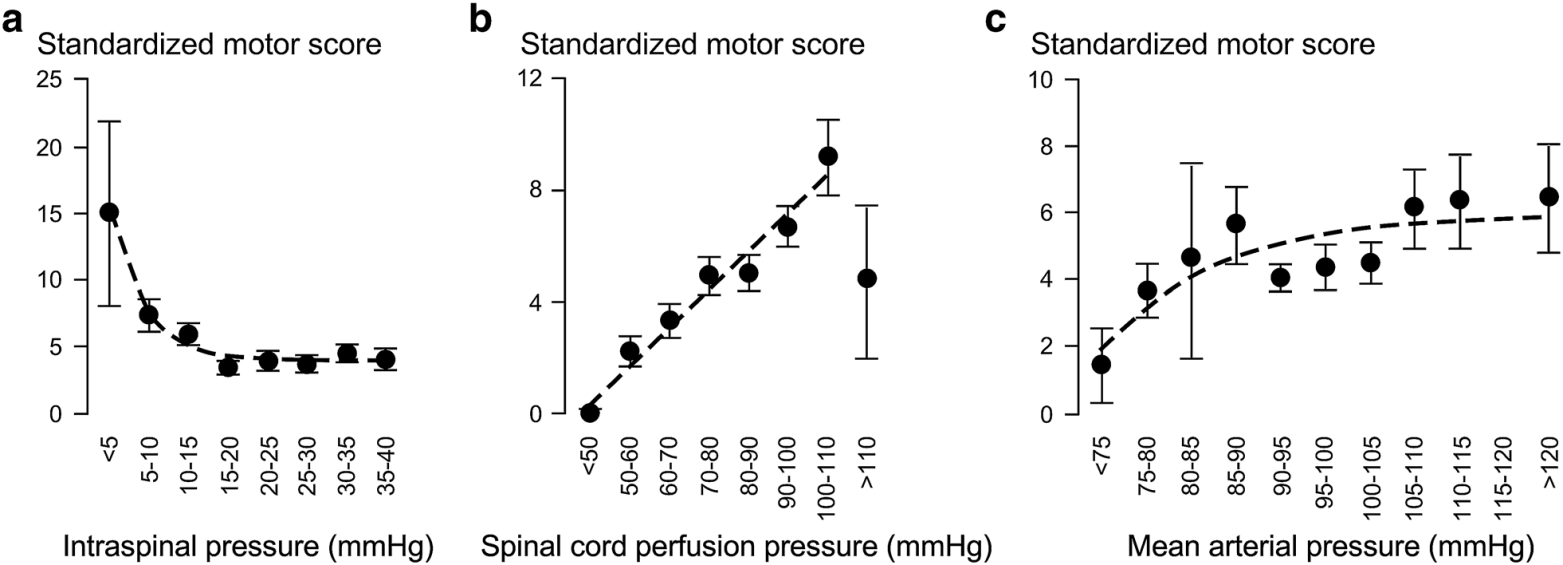
Standardized motor score correlates with injury site physiology. Standardized motor score versus **a** intraspinal pressure, **b** spinal cord perfusion pressure and **c** mean arterial pressure. Mean ± standard error. Trends (dotted gray line) modeled as exponential decay (intraspinal pressure, *R*^2^ = 0.98, *P* < 0.0005), linear (spinal cord perfusion pressure in the range < 50 to 110 mmHg, *R*^2^ = 0.96, *P* < 0.0005), and bounded exponential (Mean arterial pressure, *R*^2^ = 0.73, *P* < 0.05)

Figure 2 shows the relations between standardized motor score versus ISP, versus SCPP, and versus MAP using data from all 19 patients. The curve $y=3.9+33.4{e}^{-1.1x}$ fits well with the relation between average motor score (*y*) versus ISP class (*x* = 1, …, 8, respectively, defined as < 5, …, 35 – 40 mmHg). Reduction in ISP from > 20 to < 5 mmHg is associated with an average gain of about 11 motor points, whereas fluctuations in ISP in the range 20–40 mmHg are not associated with changes in average motor score. The line $y=1.4x-3.9$ fits well with the relation between average standardized motor score (*y*) versus SCPP class (*x* = 1, …, 7, respectively, defined as 40–50, …, 100–110 mmHg). Increasing SCPP from < 50 mmHg, up to 110 mmHg, is associated with an average gain of about 8–9 motor points. Increasing SCPP beyond 110 mmHg is associated with a reduction in motor score. The curve $y=-0.1+6.0(1-{e}^{-0.4x})$ fits well with the relation between average standardized motor score (*y*) versus MAP class (*x* = 1, …, 11, respectively, defined as < 75, …, > 120 mmHg). Increase in MAP from < 75 to 95 mmHg is associated with an average gain of about 4 motor points, whereas fluctuations in MAP in the range 95–130 mmHg are not associated with changes in average standardized motor score.

### Motor Score Strongly Correlates with Injury Site Metabolism

**Fig. 3:**
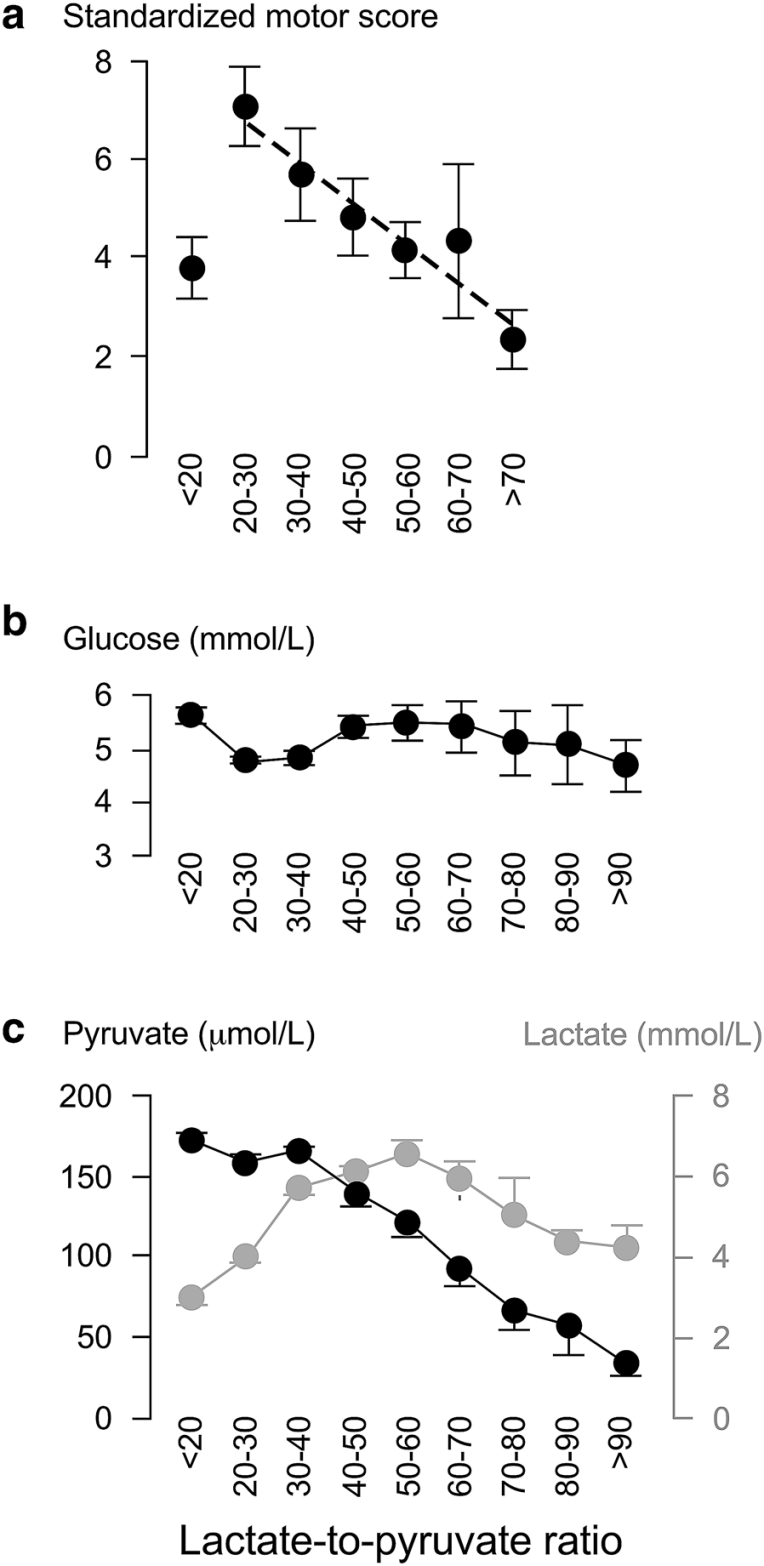
Standardized motor score correlates with injury site metabolism. **a** Relation between standardized motor score and lactate-to-pyruvate ratio. **b** Glucose and **c** Lactate + Pyruvate versus lactate-to-pyruvate ratio. Mean ± standard error. In a, the dotted line is the best fit straight line for lactate-to-pyruvate ratio in the range 20 to > 70 mmHg, *R*^2^ = 0.92, *P* < 0.005

Figure 3 shows that the line $y=-0.8x+8.3$ fits well with the relation between average standardized motor score (*y*) versus LPR class (*x* = 2, …, 6, respectively, defined as 20–30, …, > 70), plotted using data from the 13 patients who had MD monitoring. Reducing the LPR from > 70 to 20–30 is associated with an average gain of about 4 motor points. Reducing the LPR below 20 is associated with a reduction in average standardized motor score. Increasing the LPR from 20–30 to 30–40 was associated with no change in pyruvate but increase in lactate. As LPR increases beyond 50, both pyruvate and lactate drop.

### Injury Site Physiology and Metabolism Are Closely Related

**Fig. 4:**
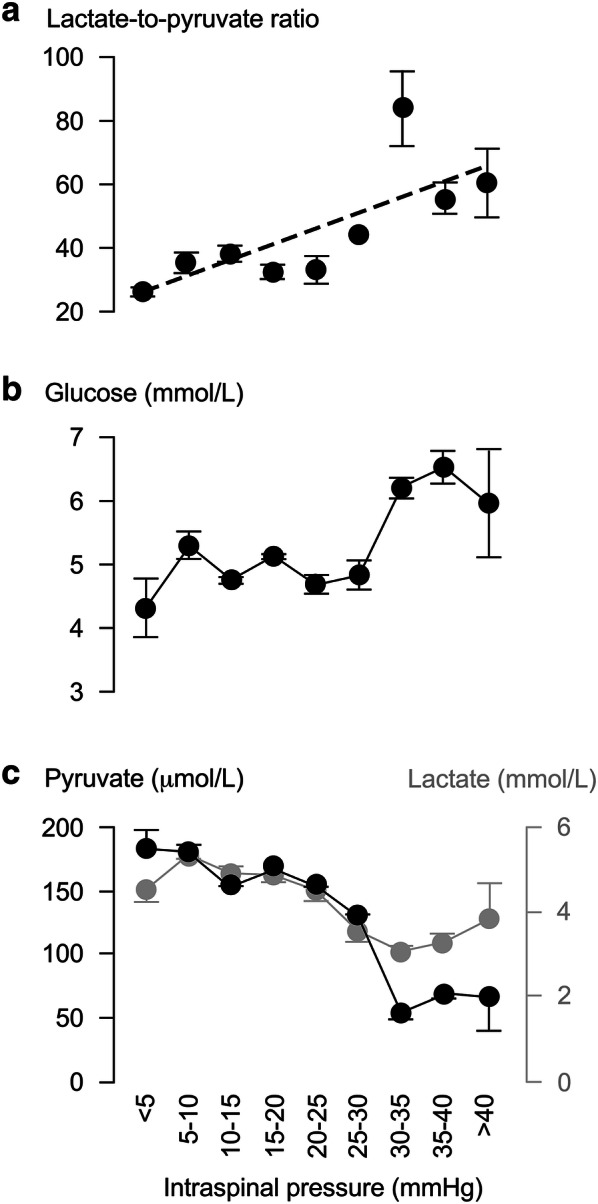
Intraspinal pressure correlates with injury site metabolism. **a** Tissue glucose, **b** tissue lactate-to-pyruvate ratio and **c** tissue pyruvate (black) + lactate (gray), versus Intraspinal pressure. Mean ± standard error. In a, best fit straight line *R*^2^ = 0.57, *P* < 0.05

**Fig. 5:**
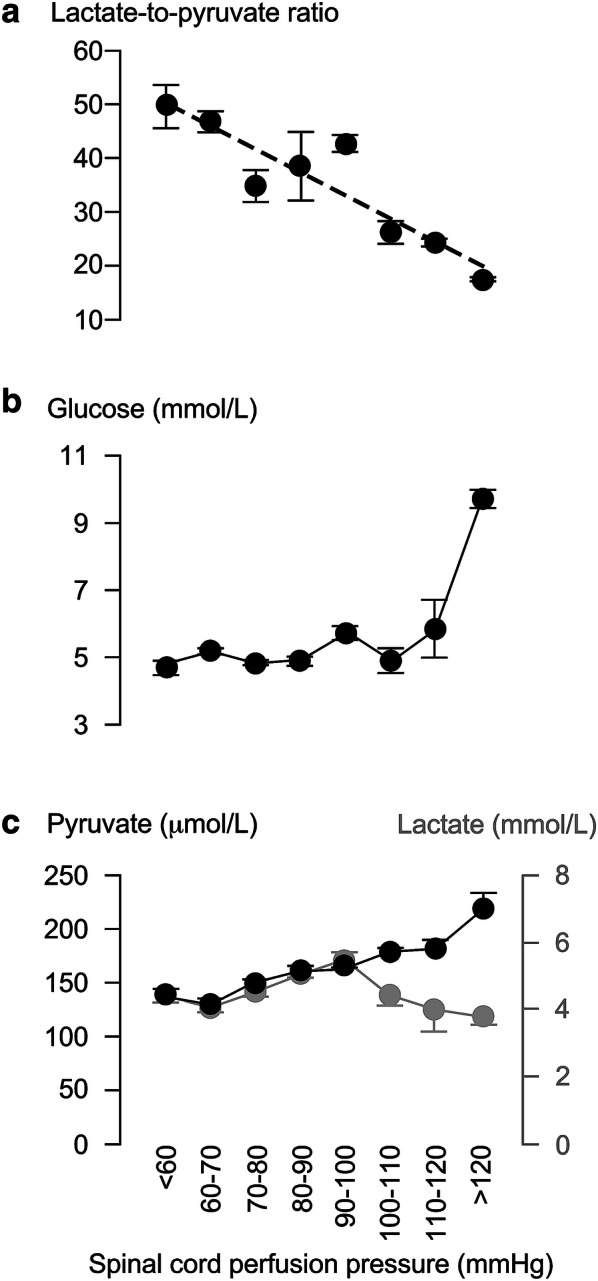
Spinal cord perfusion pressure correlates with injury site metabolism. **a** Tissue glucose. **b** Tissue lactate-to-pyruvate ratio and c. tissue pyruvate (black) + lactate (gray), versus ISP. Mean ± standard error. In a, best fit straight line *R*^2^ = 0.83, *P* < 0.005

Figure 4 shows how ISP is related to injury site metabolism. LPR (*y*) and ISP class (*x* = 1, …, 9, respectively, defined as < 5, …, > 40 mmHg) positively correlate according to $y=5.0x+20.5$. As ISP increases, glucose increases, whereas lactate and pyruvate fall. Figure 5 shows how SCPP is related to injury site metabolism. LPR (*y*) and SCPP class (*x* = 1, …, 8, respectively, defined as < 60, …, > 120 mmHg) negatively correlate according to $y=-4.3x+54.3$. As SCPP increases up to 110 mmHg, glucose remains constant, whereas lactate and pyruvate slightly increase. As SCPP increases beyond 110 mmHg, glucose and pyruvate rise, whereas lactate falls.

### Causality Analysis

**Fig. 6:**
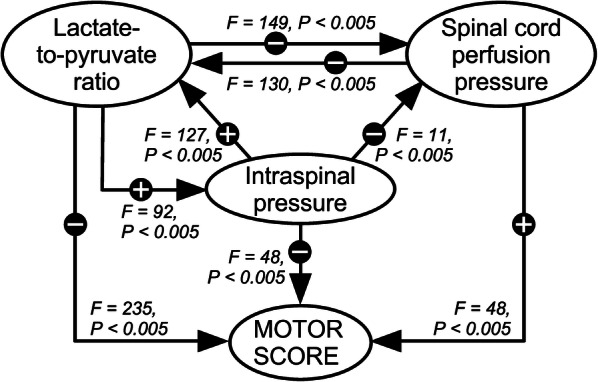
Granger causality relations. Each arrow indicates the direction of information flow, i.e., causal influence, with corresponding *F* and *P* values. ‘ + ’ or ‘–’ indicate the correlation between the variables. Causality arrows are shown if *P* < 0.05. Analysis is shown for lag = 1, but also holds for lag = 2 or 3. For details, see supplement

Granger-causal relations between the motor score, ISP, SCPP, and LPR time series are illustrated in Fig. 6. Increasing LPR Granger-causes increased ISP, decreased SCPP, and decreased motor score. In other words, worse injury site metabolism increases cord swelling, reduces cord perfusion, and increases limb weakness. Increasing SCPP Granger-causes decreased LPR and increased motor score. In other words, improving spinal cord perfusion improves spinal cord metabolism and improves limb weakness. Increasing ISP Granger-causes increased LPR, decreased SCPP, and decreased motor score. In other words, increased cord swelling reduces cord perfusion, worsens cord metabolism, and increases limb weakness.

## Discussion

### Key Results

We showed that, after spinal cord injury, fluctuations in injury site physiology (cord edema, cord perfusion) and metabolism (LPR) Granger-cause fluctuations in limb power. These causal relations between events at the injury site and limb power were established by monitoring patients with acute, motor-incomplete spinal cord injuries.

### Limitations

Though the study has a relatively small number of patients (19), our conclusions are supported by a large amount of monitoring data including 2,306 h of ISP and SCPP monitoring, 1,703 h of MD monitoring and 366 motor examinations. Another issue is that causality is a deeply philosophical concept with many possible answers that do not satisfy everyone, compared with our definition of causality, which is purely mathematical.

### Granger Causality

Establishing causation in medicine is based on the totality of evidence that includes strong association, biological mechanism, consistent finding, temporal sequence, and dose–response [33]. In the absence of a randomized trial, which is considered the “gold standard” to establish causation [34, 35], we investigated causal relations in our observational data using Granger analysis [27, 28]. Granger’s definition of causality, initially developed for financial forecasting, satisfies two intuitive notions: First, the cause always precedes the effect. Second, the cause carries unique information about the future effect, i.e., there is information flow from the past of the cause to the future of the effect that cannot be accounted for by the past of the effect. Implicit in these notions is that intervention to alter the cause should result in a predictable change in the future of the effect. Advantages of Granger are that it is expressed in relatively straightforward mathematics that permit hypothesis testing and provide information about the strength of each causality [34, 35]. Alternative analyses that can distinguish between causation and correlation may be performed, e.g., convergent cross mapping, which is based on complexity theory and, unlike Granger, does not assume linear relations [36].

### Interpretation of Correlations

Multiple correlations between injury site physiology, metabolism, and neurological status were defined. Here, we highlight some interesting findings: First, optimal ISP is less than 5 mmHg, i.e., no cord compression. Second, increase in ISP from 20 to 40 mmHg is associated with increase in LPR, but no reduction in limb power, probably because the standardized motor score is already very low. Third, as SCPP increases, LPR decreases (less ischemia) and limb power improves but, beyond 110 mmHg, limb power decreases, suggesting that hyper-perfusing the injury site is detrimental. Metabolically, hyper-perfusion is characterized by very low (less than 20) LPR and high glucose; though this is aerobic metabolism, our data suggest that the high glucose is detrimental as previously reported [37–39] due to increased endoplasmic reticulum stress, inflammation and free radicals. Fourth, increasing the MAP beyond 85–90 mmHg, which remarkably corresponds to the recommended target [40], does not improve limb power. A possible explanation is that low MAP indicates injury site ischemia regardless of ISP, but above 90 mmHg, the same MAP corresponds to injury site ischemia in some patients and hyper-perfusion in others. In practical terms, increasing the SCPP improves limb power more than increasing the MAP. Fifth, the correlations between ISP and SCPP versus limb power are reminiscent of similar relations versus neurological improvement at 9–12 months [20]. Sixth, though there is inverse correlation between LPR and limb power, LPR below 20 appears detrimental likely related to hyper-perfusion state described above. Seventh, as LPR increases up to 50–60, pyruvate decreases and lactate increases (indicating compensatory switch from aerobic to anaerobic metabolism at the injury site), but as LPR increases beyond 60, both pyruvate and lactate decrease (indicating overall suppression of aerobic and anaerobic metabolism). Though the findings described here provide a unique insight into how spinal cord physiological and metabolic events relate to limb power, they are associations that do not imply causations.

### Interpretation of Causal Effects

The causal relations between LPR, SCPP, ISP, and standardized motor score in Fig. 6 make biological sense. A metabolic insult (high LPR, e.g., from hypoxia or infection) Granger-causes cord swelling (high ISP), Granger-causes reduction in cord perfusion, and Granger-causes impaired neuronal function (reduced motor score). Increase in spinal cord perfusion Granger-causes increase in aerobic metabolism at the injury site (decreased LPR) and Granger-causes the motor score to improve. Cord swelling (increase in ISP) Granger-causes more anaerobic metabolism (LPR increases), Granger-causes drop in SCPP (= MAP – ISP) and Granger-causes reduction in limb power. Interestingly, Granger analysis did not produce absurdities, e.g., change in limb power Granger-causes physiological or metabolic changes at the injury site. A key finding in Fig. 6 is several positive feedback loops (vicious cycles), e.g., increased LPR Granger-causes increased cord swelling, in turn Granger-causing increase in LPR. Multi-step vicious cycles are also evident, e.g., increased LPR Granger-causes increased cord swelling, which Granger-causes decreased SCPP, in turn Granger-causing increase in LPR. The presence of so many vicious cycles implies that insults to the injury site (e.g., hypoxia, acidosis, etc.) are amplified, thus emphasising the importance of treating such insults to prevent secondary cord damage.

### Implications for Treatment

ISP is increased by cord compression from bone fragments, hematoma, and dura. Decompression of the injured cord including duroplasty would reduce ISP [41]. MAP may be increased using vasopressors [30]. Reducing ISP and increasing MAP will, in turn, increase SCPP. Interventions to reduce LPR (other than reducing ISP and increasing SCPP) include management of hypoxia, fever, and acidosis (e.g., limiting blood loss during surgery) [21, 32].

### Generalizability

To study the relations between physiological/metabolic events at the injury site and limb power, we focused here only on AIS C TSCI patients. Our key conclusion that reducing ISP, increasing SCPP, and reducing LPR improve neurological status is likely to apply to all injury severities. Future studies may explore this further, e.g., by determining the effect of increasing SCPP on sensory level in AIS A and B TSCI patients.

## Conclusions

The causal relationships described here suggest that interventions to normalize LPR, SCPP, and ISP are likely to improve limb power in AIS C TSCI patients.

## Electronic supplementary material

Below is the link to the electronic supplementary material.

Supplementary file1 (PDF 26 kb)
