# Supplementary material for: Acute Spinal Cord Injury: Correlations and Causal Relations Between Intraspinal Pressure, Spinal Cord Perfusion Pressure, Lactate-to-Pyruvate Ratio, and Limb Power
Source: Neurocrit Care. 2020 May 20;34(1):121–9. doi: 10.1007/s12028-020-00988-2 (PMC7940162; doi:10.1007/s12028-020-00988-2)
Supplement: Supplementary file 1 — Supplementary file1 (PDF 26 kb) [file 12028_2020_988_MOESM1_ESM.pdf]

SUPPLEMENT: GRANGER CAUSALITY ANALYSIS

The following explains how we can test whether two time-series are causally related according to Granger. This analysis was used to obtain Fig. 6.

Suppose we want to check whether SCPP causes MS.

Spinal cord perfusion pressure (SCPP = X) and motor score (MS = Y) are time-series such that:

$X = (X_1, X_2, \ldots, X_N)$, and $Y = (Y_1, Y_2, \ldots, Y_N)$.

First, we try to predict the Y values using earlier (lagged) Y values, i.e. we fit the following linear regression:

$$Y_t = a_{10} + \sum_{i=1}^{m} a_{1i} Y_{t-i} + \varepsilon_{1t}$$

which produces ESS_1 as the sum of squared residuals (i.e. differences between predicted and actual Y_t).

Then, we predict the Y values using not only lagged Y values buy also lagged X values, i.e. we fit the following linear regression:

$$Y_t = a_{20} + \sum_{i=1}^{m} a_{2i} Y_{t-i} + \sum_{j=1}^{k} \beta_j X_{t-j} + \varepsilon_{2t}$$

which produces ESS_2 as the sum of squared residuals (i.e. differences between predicted and actual Y_t).

In these equations, $(a_{11}, a_{12}, \ldots, a_{1m})$ and $(a_{21}, a_{22}, \ldots, a_{2m})$ are the coefficients of the lagged values of Y_t , $(\beta_1, \beta_2, \ldots, \beta_k)$ are the coefficients of the lagged values of X_t , ε_{1t} and ε_{2t} are white noises, and m and k are lag lengths.

The null hypothesis, i.e. X does not Granger-cause Y, is:

$$H_0: \beta_1, \beta_2, \ldots, \beta_k = 0$$

In simple terms this means that the prediction of Y based on past values of Y is no better than the prediction of Y based on past values of both X and Y. The null hypothesis can be tested using the F statistic, computed as:

$$F = \frac{(ESS_1 - ESS_2)/k}{(ESS_2/(N-k-m-1)}$$

We then compare he F statistic to a critical value to obtain a *P*-value, which determines whether to accept or reject the null hypothesis.

The process is then repeated, to check whether MS causes SCPP or to test causality between other time series e.g. ISP *versus* MS or LPR *versus* MS etc.
